# Altered structural and functional homotopic connectivity associated with cognitive changes in SLE

**DOI:** 10.1136/lupus-2024-001307

**Published:** 2024-11-24

**Authors:** Sha Ni, Ning An, Chunlei Li, Yue Ma, Pengfei Qiao, Xueying Ma

**Affiliations:** 1 Inner Mongolia Medical University, Hohhot, Inner Mongolia, China; 2 Department of Rheumatology, Affiliated Hospital of Inner Mongolia Medical University, Hohhot, Inner Mongolia, China; 3 Department of Radiology, Inner Mongolia Cardiovascular and Cerebrovascular Hospital, Hohhot, Inner Mongolia, China; 4 Department of Radiology, Affiliated Hospital of Inner Mongolia Medical University, Hohhot, Inner Mongolia, China

**Keywords:** Systemic Lupus Erythematosus, Magnetic Resonance Imaging, Antibodies, Autoimmune Diseases

## Abstract

**Objective:**

Previous studies have revealed functional changes within the cerebral hemispheres of patients with SLE; however the changes between cerebral hemispheres are still unknown. The present study aimed to explore the functional and structural changes between bilateral hemispheres using functional MRI and find their relationship with cognition in patients with SLE.

**Methods:**

54 patients with SLE and 32 age-matched and sex-matched healthy controls (HCs) underwent MRI scanning and neuropsychological testing, and clinical data was collected in patients with SLE. Voxel-mirrored homotopic connectivity (VMHC) values and grey matter volume were calculated for all subjects. Correlation analysis was established to determine the relationship between VMHC values, grey matter volume and cognitive scores, blood biochemical markers in patients with SLE.

**Results:**

Compared with HCs, patients with SLE showed increased VMHC values in the insula and parahippocampal gyrus, while grey matter volume were reduced in these regions. Correlation analysis demonstrated that the increased VMHC values in insula was negatively correlated with decreased orientation function and positively correlated with decreased attention function. The grey matter volume in insula was negatively correlated with decreased attention and abstraction. The VMHC values and grey matter volume in insula and parahippocampal gyrus were negatively associated with lupus-specific antibodies.

**Conclusion:**

The structural and functional changes of insula and parahippocampal gyrus might be potential neuroimaging markers, and specific antibodies associated with lupus might be involved in the pathophysiological mechanisms of brain dysfunction.

**Trial registration number:**

NCT06226324.

**WHAT IS ALREADY KNOWN ON THIS TOPIC**

- Patients with SLE may have abnormalities in functional activity between or within the cerebral hemispheres, but the relationship between the synergy between the cerebral hemispheres, grey matter volume and cognitive function have not been fully clarified yet.

**WHAT THIS STUDY ADDS**

- Voxel-mirrored homotopic connectivity (VMHC) values and grey matter volume in patients with SLE were associated with cognitive function.
- Patients with SLE displayed VMHC values and grey matter volume in the insula and parahippocampal gyrus were associated with lupus-related specific antibodies.

**HOW THIS STUDY MIGHT AFFECT RESEARCH, PRACTICE OR POLICY**

- This study may help to understand the functional and structural characteristics of brain injury in patients with SLE, and autoimmune antibodies might be involved in the pathogenesis.

## Introduction

 SLE is a recurrent autoimmune disease of variable clinical manifestations, characterised by multiple organs and systems involvement.^1 2^ Neuropsychiatric SLE (NPSLE) is one of the most common complications of SLE, which can occur in the early stages of disease course, accompanied by a series of symptoms involving the central and peripheral nervous systems. Cognitive dysfunction is one of the most common neurologic and psychiatric manifestations, with a prevalence up to 80% in patients with NPSLE.^3^ NPSLE is the second leading cause of death after lupus nephritis, and the mortality of NPSLE is tripled when compared with patients with SLE without neuropsychiatric symptoms.^4 5^ However, the physiopathologic mechanism of NPSLE is still unclear. More and more researches support the view that both antibody-mediated neuroimmune interface injury and ischaemia are contributing to the mechanism of neuronal damage in NPSLE.^4^ Hirohata *et al* showed that the level of anti-Sm antibodies in cerebrospinal fluid of patients with NPSLE correlated with the degree of blood–brain barrier damage and found that anti-Sm could bind to the surface of certain neurons.^6^ In addition, a cohort study revealed that anti-phospholipid (anti-aPL) antibodies were associated with ischaemic brain changes such as lacunar infarcts in patients with SLE.^7^ Early diagnosis is crucially important for improving the prognosis of patients with NPSLE, but currently the diagnosis of NPSLE mainly relies on non-specific neuropsychiatric symptoms. So far, researchers have tried to use MRI technology to explore structural and functional changes of brain in patients with SLE. Previously, a study using MRI structural technique showed that patients with NPSLE demonstrated a correlation between brain volume, white matter hyperintensity volume and cognitive functions.^8^ Functional MRI (fMRI) can provide more information of early cerebral changes such as blood flow, fibre tracts and functional connections, which is widely used in the brain science.

The brain is a complex network centre for information processing. Cognition is formed in cerebral cortex by separating and integrating the information,^9^ which requires the cooperation between different regions within and inter the cerebral hemispheres. Voxel-mirrored homotopic connectivity (VMHC) is an effective method that can quantitatively evaluate the homotopic connectivity between each voxel in one hemisphere and the corresponding voxel in the mirror hemisphere. VMHC is based on the resting-state fMRI to assess brain activity changes,^10^ which is widely used in the neurodegenerative and psychiatric diseases. Recently, Chen *et al* found that compared with stable mild cognitive impairment (MCI) patients, progressive MCI patients displayed significantly decreased VMHC in the insula and thalamus and reduced volume of the corpus callosum.^11^ Further analysis demonstrated that decreased VMHC was related to atrophy of the corpus callosum and was directly related to cognitive dysfunction,^11^ indicating that changes in VMHC played an important role in the pathogenesis of cognitive dysfunction. As for psychiatric disorders, a study that focused on melancholic and non-melancholic major depressive disorder showed significantly decreased VMHC in the precentral gyrus of melancholic patients,^10^ suggesting that VMHC in the precentral gyrus might be a potential imaging marker to distinguish disease states and involved in the neural mechanism of brain damage. However, few studies focus on the application of VMHC in brain injury of patients with SLE. This study aimed to explore the changes of VMHC and grey matter volume in patients with SLE and find the relationship between brain dysfunction with cognitive function. We hypothesised that there are different damage patterns in interhemispheric functional connectivity (FC) of patients with SLE accompanied by grey matter changes, aiming to find potential neuropathological mechanisms of brain injury in SLE.

## Materials and methods

### Participants

54 patients with SLE were recruited from the department of Rheumatology and Immunology of the Affiliated Hospital of Inner Mongolia Medical University, China, from January 2022 to August 2023. The inclusion criteria for all patients with SLE was as follows: (1) fulfilling the revised SLE diagnostic criteria of the American College of Rheumatology in 2019^12^; (2) women; (3) aged 18–55 years; (4) right handed; and (5) cooperating with neuropsychological testing and MRI examination. The exclusion criteria was as follows: (1) concomitant other autoimmune diseases; (2) concomitant other neurological/psychiatric diseases; (3) history of brain tumours, haemorrhages, infarctions or other diseases; and (4) history of drug abuse or alcohol abuse. 32 gender-matched and age-matched healthy controls (HCs) were recruited from the community. The inclusion criteria for HCs included: (1) women; (2) aged 18–55 years; (3) right handed; (4) able to cooperate with MRI examinations and neuropsychological testing; (5) no history of neurological/psychiatric diseases or other autoimmune diseases; (6) no structural brain abnormalities such as cerebrovascular disease or brain tumour; (7) no abuse of drugs or alcohol; and (8) no diabetes, hypertension and other risk factors (online supplemental file 1). It has been successfully registered in the U.S. Clinical Trials Database (NCT06226324, https://classic.clinicaltrials.gov/). All the patients with SLE and HCs provided written informed consent.

### Demographic and clinical data

Demographic data was collected for all subjects. For patients with SLE, clinical data such as disease duration and SLE Disease Activity Index 2000 (SLEDAI-2K) were recorded. Laboratory variables including red blood cell, haematocrit, haemoglobin, C reactive protein, erythrocyte sedimentation rate, anti-β2GP1 antibodies (anti-β2GP1), anti-nucleosome antibodies, anti-dsDNA, anti-cardiolipin antibodies, C3 and C4, lupus anticoagulants LA1 and LA2 levels were also recorded. All data for SLE were obtained from clinical history (table 1).

**Table 1:** Demographic and clinical characteristics of the participants

	SLE (n=54)	HC (n=32)	Statistical quantity	P value
Age (years)	39.09±10.16*	38 (20.5)†	−0.921	0.357
Education (years)	12 (6)†	16 (5)†	−3.134	**0.002‡**
Disease duration (months)	60 (149.75)†	N/A	N/A	N/A
SLEDAI-2K	9.5 (8.25)†	N/A	N/A	N/A
ESR (mm/hour)	17 (31.5)†	N/A	N/A	N/A
CRP (μg/L)	1.07 (2.85)†	N/A	N/A	N/A
Hb (g/L)	120.67±17.32*	N/A	N/A	N/A
RBC (10^12^/L)	4.05±0.53*	N/A	N/A	N/A
HCT (%)	37.08±5.36*	N/A	N/A	N/A
Anti-β2GP1 (RU/mL)	4.86 (4.98)†	N/A	N/A	N/A
ANuA (RU/mL)	12.59 (69.5)†	N/A	N/A	N/A
Anti-dsDNA (U/mL)	66.85 (333.46)†	N/A	N/A	N/A
aCL (U/mL)	12.52 (19.35)†	N/A	N/A	N/A
C3 (g/L)	0.70 (0.44)†	N/A	N/A	N/A
C4 (g/L)	0.13 (0.15)†	N/A	N/A	N/A
LA1 (s)	36.05 (10.4)†	N/A	N/A	N/A
LA2 (s)	31.05 (3.37)†	N/A	N/A	N/A

The Pp value was obtained by Mann–-Whitney U Ttest. Bold values indicate statistical significance.

* Values are expressed as means (standard deviation, SD).

† Values are expressed as median, with interquartile rangeIQRs in parentheses.

‡ p<0.05.

aCLanti-cardiolipin antibodiesanti-β2GP1anti-beta-2-glycoprotein 1 antibodyANuantinucleosome antibodyC3/C4complementCRPC reactive proteinESRerythrocyte sedimentation rateHbhaemoglobinHChealthy controlsHCThaematocritLA1/2lupus anticoagulantNAunable to obtain valid valuesRBCred blood cell countSLEDAI-2KSLE Disease Activity Index 2000

### Neuropsychological assessment

All subjects underwent a neuropsychological evaluation before MRI scanning, including the Trail Making Test (TMT) and the Beijing version of the Montreal Cognitive Assessment (MoCA). The TMT^13^ is used to assess attention and executive function, requiring subjects to connect randomly arranged numbers in sequence as quickly as possible, while the operator records time. The MoCA^14 15^ Scale is used to assess overall cognitive function, including eight cognitive domains such as attention and concentration, executive function, memory, language, visuospatial constructional skills, abstract thinking, calculation and orientation, with a total score of 30 points. A final score of ≥26 is considered normal, while a score of <26 indicates cognitive impairment (CI). If the subject has less than 12 years of education, an additional one point is added, with a total score not exceeding 30 points.

### MRI data acquisition

All subjects underwent MRI scans using a 3.0-Tesla Siemens Skyra scanner (Germany) with a 20-channel phased-array head coil. T1-weighted anatomical images were obtained using a volumetric three-dimensional magnetisation prepared by rapid gradient-echo sequence with the following parameters: repetition time (TR)=2300 ms, time echo (TE)=2.32 ms, flip angle=8°, field of view (FOV)=240×240 mm^2^, slice thickness=0.9 mm, matrix=256×256, voxel size=0.9×0.9×0.9 mm^3^ and slices=192. Blood oxygen level dependent (BOLD) functional images were acquired using an echo-planar imaging sequence with the following parameters: TR=2000 ms, TE=30 ms, flip angle=90°, FOV=224×224 mm, slice thickness=2 mm, no slice gap, matrix=64×64, voxel size=3.5×3.5×4.2 mm^3^ and a total of 190 time points.

### Preprocessing of structural MRI

Preprocessing of structural MRI images was conducted using MATLAB R2018a (https://www.mathworks.cn/en/products/matlab.html) with the SPM V.12 (https://www.fil.ion.ucl.ac.uk/spm/) and CAT V.12 (https://www.nitrc.org/projects/cat/) toolboxes. The Digital Imaging and Communications in Medicine (DICOM) raw image files were converted to Neuroimaging Informatics Technology Initiative (NIfTI) format and bias field correction was performed. Subsequently, all images were segmented to obtain volume of grey matter, white matter, cerebrospinal fluid. The images were registered to the Montreal Neurological Institute (MNI) space using the Diffeomorphic Anatomical Registration Through Exponentiated Lie Algebra (DARTEL) algorithm. Subjects with an average score below B+ in the image quality report were excluded. The grey matter images were then smoothed using a Gaussian kernel with a full width at half maximum (FWHM) of 6×6×6 mm.

### Preprocessing of resting-state fMRI

The images were preprocessed using DPARSF in the MATLAB R2018a platform. The preprocessing steps included converting the DICOM raw image files to NIfTI format, removing the first 10 time points and correcting for slice timing and head motion. Subjects with head motion exceeding 2° rotation or 2 mm translation in any direction were excluded. The average frame displacement (FD) was calculated, and images with FD>0.5 were removed. Each T1 structural image was coregistered with the corresponding functional image after correction and segmented into grey matter, white matter and cerebrospinal fluid. The functional images were normalised to the standard MNI space and resampled to 3×3×3 mm^3^ voxel size. Linear regression was used to remove the effects of covariates such as white matter, cerebrospinal fluid signal and head motion parameters. The images were smoothed with a Gaussian kernel with a FWHM of 6 mm. Finally, linear drift was removed, and a band-pass filter of 0.01–0.08 Hz was applied. The DPABI toolbox was applied to perform VMHC analysis. We calculated the homotopic FC between each voxel in one hemisphere of the brain and its mirrored counterpart in the other hemisphere, both registered to the MNI space, using the Pearson correlation coefficient. The resulting correlation coefficients were then transformed using Fisherr’s transformation to improve normality.

Data collection of all subjects was operated by an experienced radiologist and head was strictly fixed before scanning. During the data analysis, we did not find any subject with an average score below B+, average FD more than 0.5 or the head movement greater than 2°or 2 mm. Thus, all subjects met the image quality control standards, and all subjects were included in the subsequent analysis.

### Statistical analysis

Demographic data and difference in neuropsychological scores between two groups were compared, using SPSS V.20.0 software (https://www.ibm.com/cn-zh/products/spss-statistics). All continuous variables were tested for normality using Shapiro-Wilk test. Normally distributed variables were presented as mean±SD $\left(\overset{-}{x}\pm s\right)$, while non-normally distributed variables were presented as median (IQR). Mann-Whitney U test was used to analyse differences in age and education level between SLE and HC groups. Analysis of covariance was used to compare differences in neuropsychological scores across two groups, and p<0.05 was considered statistically significant.

We analysed the differences in VMHC and whole-brain grey matter volume between the two groups using the SPM V.12 toolbox. Correlation analysis was performed between neuropsychological scores and whole-brain grey matter volume or VMHC in SLE group. Age, education and total intracranial volume (for grey matter volume) were included as covariates in the regression analysis. We applied Gaussian Random Field (GRF) correction, as a voxel-level threshold of p<0.005 and a cluster-level threshold of p<0.05 to determine statistical significance. All differential and correlation results were displayed using DPABI.

## Results

### Demographic and clinical characteristics

A total of 86 subjects were recruited in this study, including 54 patients with SLE and 32 HCs. The mean or median age of SLE and HC groups was 39 (39.09±10.16) and 38 years, respectively, and no statistically difference was observed (p>0.05). The median education level of SLE and HC groups was 12 and 16 years, respectively, which had statistically difference (p<0.05). SLE group had a median disease duration of 60 months, with the median SLEDAI-2K scores of 9.5 points. The laboratory biochemical markers collected from the SLE group are shown in table 1.

### Neuropsychological assessment

Due to the statistical difference in education level between SLE and HC groups, which might affect cognition, we employed covariance analysis to compare the differences in neuropsychological assessment scores between two groups with education level as a covariate (p<0.05 was considered statistically significant). The results displayed that patients with SLE had significantly longer TMT duration and lower score in the language domain (p<0.05) when compared with HCs. There were no significant differences in visuospatial and executive function, naming, attention, abstraction, memory, orientation and total score between patients with SLE and HCs (table 2).

**Table 2:** Neuropsychological tests with comparisons between SLE and HC groups

	SLE (n=54)	HC (n=32)	Statistical quantity	P value
TMT	50.65 (31.08)*	34.1 (20.28)*	5.546	**0.021†**
MoCA				
Visuospatial/executive function	3 (2)*	4 (1)*	0.789	0.377
Naming	3 (1)*	3 (0)*	0.01	0.919
Attention	6 (1)*	6 (0)*	0.004	0.95
Language	2 (1)*	3 (1)*	4.614	**0.035†**
Abstract	2 (1)*	2 (0)*	0.464	0.497
Memory	2 (2.25)*	4 (2)*	0.964	0.329
Orientation	6 (1)*	6 (0)*	2.420	0.124
Total score	23.06±0.84‡	27 (3.75)*	2.799	0.098

The Pp value was obtained by analysis of Ccovariance, ANCOVA. Bold values indicate statistical significance.

* Values are expressed as median, with interquartile rangeIQRs in parentheses.

† p<0.05.

‡ Values are expressed as means (SD).

HChealthy controlsMoCAMontreal Cognitive AssessmentTMTTrail Making Test

### Comparison of VMHC values and grey matter volume between SLE and HC groups

**Figure 1:**
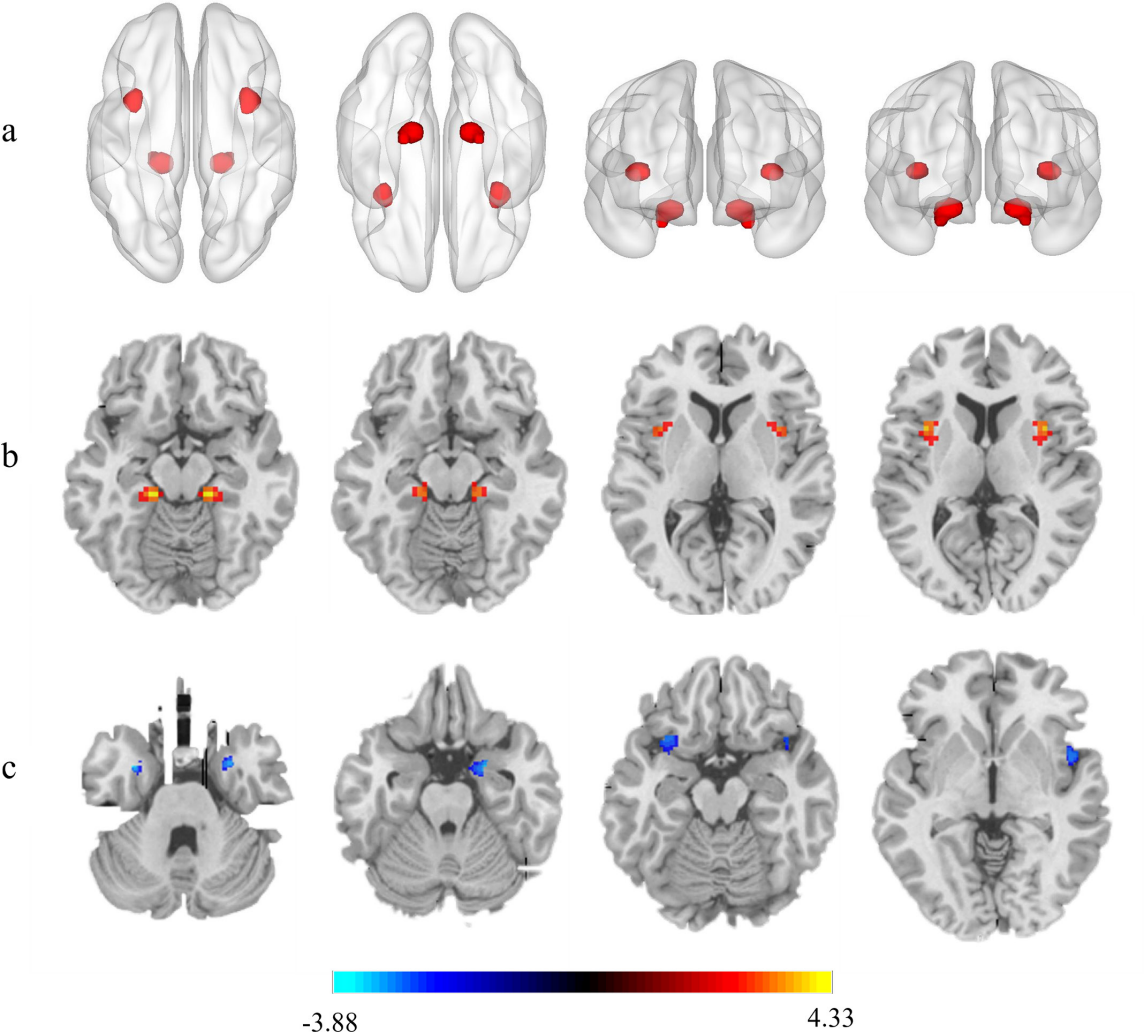
VMHC and grey matter volume differences between SLE and HC groups. (a,b) The yellow-red colour regions referred that patients with SLE demonstrated increased VMHC values in the insula and parahippocampal gyrus compared with HCs. (c) The blue regions referred that patients with SLE demonstrated decreased grey matter volume in bilateral insula and parahippocampal gyrus compared with HCs. All results controlled for age, education and total intracranial volumes as covariates. Gaussian Random Field corrected, voxel level, p<0.005; cluster level, p<0.05. HC, healthy controls; VMHC, voxel-mirrored homotopic connectivity.

Comparison of whole-brain VMHC between SLE and HC groups showed that the differential brain regions were concentrated in insula and parahippocampal gyrus. Compared with HCs, patients with SLE demonstrated increased VMHC values in the insula and parahippocampal gyrus (GRF corrected, p<0.05). Based on the statistical difference results of VMHC analysis in the two groups, we also compared the whole-brain grey matter volume between SLE and HC groups. Compared with HCs, patients with SLE demonstrated decreased grey matter volume in bilateral insula and parahippocampal gyrus (GRF corrected, p<0.05). All results were controlled for age, education level and total intracranial volume as covariates (tables3  4 and figure 1).

**Table 3:** VMHC differences in brain between SLE and HC groups

Peak region (AAL)	Peak coordinates MNI			Cluster size (voxels)	T value*
	x	y	z		
Insula_L	−36	9	9	20	4.02677
Insula_R	36	9	9	20	4.02677
ParaHippocampal_L	−18	−30	−15	32	4.33472
ParaHippocampal_R	18	−30	−15	32	4.33472

The x, y, z coordinates are the primary peak locations in the MNI space.

* GRFGaussian Random Field corrected, cluster size>20 voxel, voxel level, pp<0.005; cluster level, pp<0.05.

AALAnatomical Automatic LabelingHChealthy controlsMNIMontreal Neurological InstituteVMHCvoxel-mirrored homotopic connectivity

**Table 4:** Grey matter volume differences in brain between SLE and HC groups

Peak region (AAL)	Peak coordinates MNI			Cluster size (voxels)	T value*
	x	y	z		
Insula_L	−25.5	21	−15	244	−2.89024
Insula_R	46.5	-3	0	282	−2.88709
ParaHippocampal_L	−30	0	−33	31	−2.89354
ParaHippocampal_R	27	7.5	−24	221	−2.88637

The x, y, z coordinates are the primary peak locations in the MNI space.

* GRFGaussian Random Field corrected, voxel level, pp<0.005; cluster level, pp<0.05.

AALAnatomical Automatic LabelingHChealthy controlsLleftMNIMontreal Neurological InstituteRright

### Correlation between neuropsychological assessment scores, VMHC values and grey matter volume in SLE group

**Figure 2:**
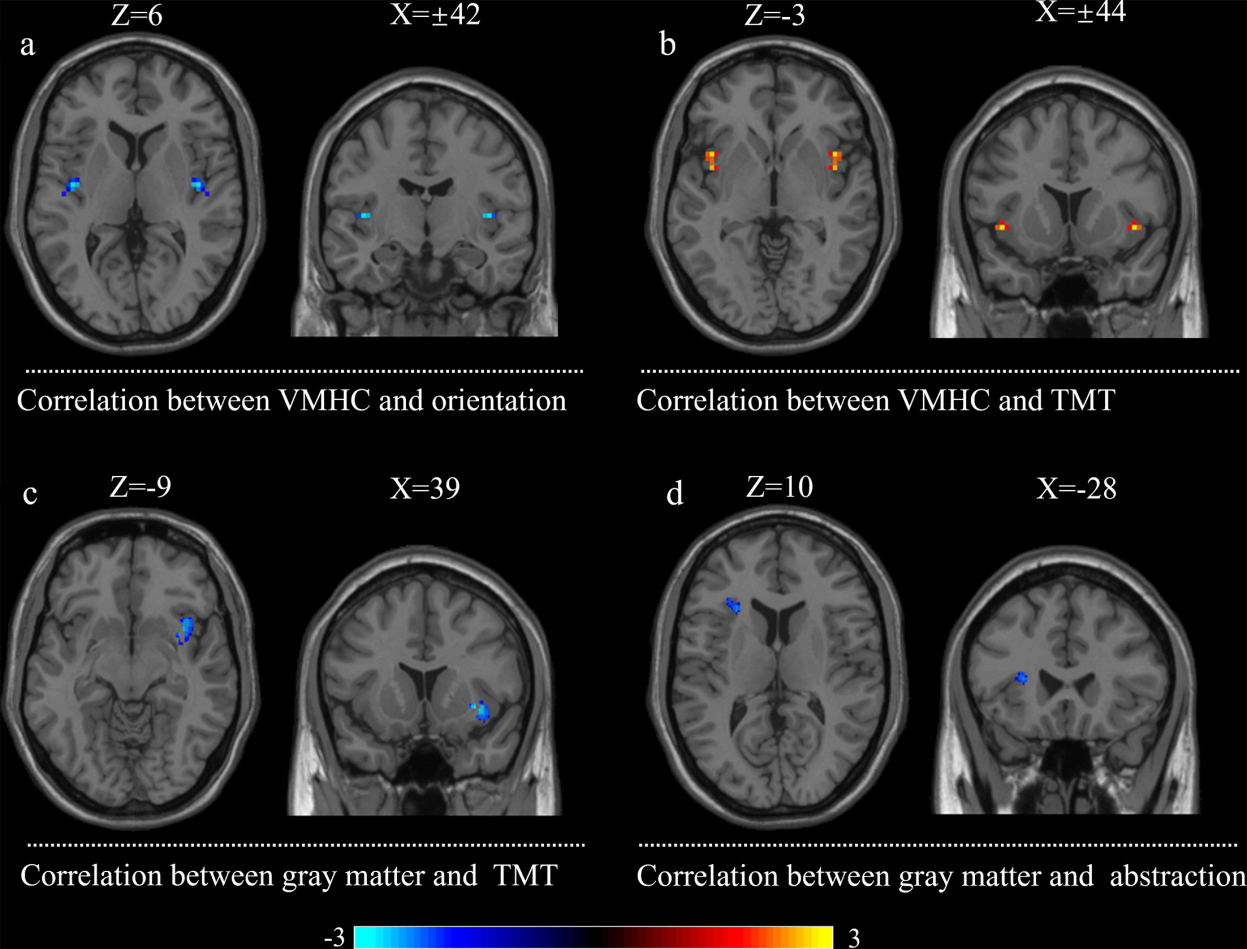
Correlation analysis between neuropsychological variables and VMHC, grey matter volume in patients with SLE. (a,b) The correlation between VMHC values and neuropsychological variables. The result respectively showed that VMHC values in the insula was negatively associated with orientation function and positively associated with the TMT duration. (c,d) The correlations between grey matter volume and neuropsychological variables. The results, respectively, showed the decreased grey matter volume in right insula was correlated with increased TMT duration, and the decreased grey matter volume in left insula was correlated with higher scores in abstraction. All results controlled for age, education, and total intracranial volumes as covariates. Gaussian Random Field corrected, voxel level, p<0.005; cluster level, p<0.05. TMT, Trail Making Test; VMHC, voxel-mirrored homotopic connectivity.

Correlation analysis showed that the increased VMHC values of the insula was negatively associated with the orientation scores, and positively associated with TMT (GRF correction, p<0.05). Moreover, the decreased grey matter volume in right insula was negatively correlated with TMT duration, and the decreased grey matter volume in left insula was correlated with higher scores in abstraction (GRF corrected, p<0.05). All results were controlled for age, education and total intracranial volume as covariates (figure 2).

### Correlation between laboratory biochemical markers, VMHC values and grey matter volume in SLE group

**Figure 3:**
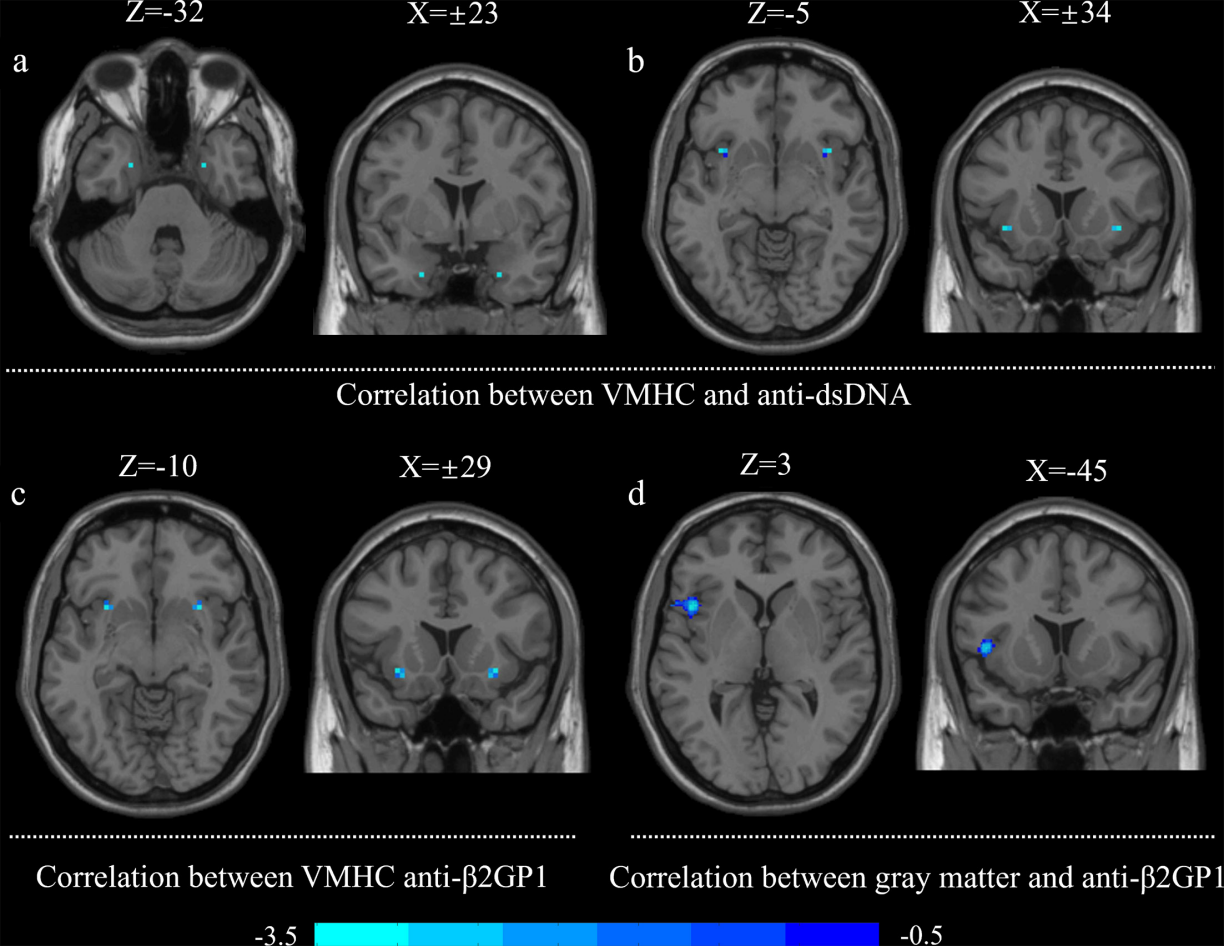
Correlation analysis between laboratory biochemical variables and VMHC values, grey matter volume in patients with SLE. (a,b) The results respectively displayed that increased VMHC values in the parahippocampal gyrus and in the insula were negatively associated with anti-ds-DNA antibodies. (c) The VMHC values in the insula was negatively correlated with anti-β2GP1 antibody. (d) The decreased grey matter volume in left insula was correlated with increased anti-β2GP1 antibody. (a–c) The correlations between VMHC values and clinical variables. (d) The correlations between grey matter volume and clinical variables. All results controlled for age, education and total intracranial volumes as covariates. GRF corrected, voxel level, p<0.005; cluster level, p<0.05. GRF, Gaussian Random Field; VMHC, voxel-mirrored homotopic connectivity.

The results displayed that VMHC values of insula was negatively associated with the level of anti-ds-DNA and anti-β2GP1 antibody, and the VMHC values in parahippocampal gyrus was negatively associated with anti-ds-DNA antibodies (GRF corrected, p<0.05). Moreover, the decreased grey matter volume in the left insula was correlated with increased anti-β2GP1 antibody; the decreased grey matter volume in the right insula was correlated with complement C4 (GRF correction, p<0.05). All results were controlled for age, education and total intracranial volume as covariates (figure 3).

## Discussion

CI is one of the main neuropsychiatric manifestations NPSLE, seriously affecting the quality of life. The purpose of this study was to explore the homotopic connectivity between cerebral hemispheres and grey matter volume changes in patients with SLE. In addition, the correlations among VMHC values, grey matter volume, laboratory biochemical markers and neuropsychological assessment scores in patients with SLE were also analysed. The results showed that patients with SLE performed worse in attention and executive function, and language, which was consistent with prior study.^16^,^18^ The VMHC and grey matter volume in the insula and parahippocampal gyrus of SLE were changed and correlated with abnormal cognition and laboratory biochemical markers such as anti-ds-DNA antibody, anti-β2GP1 antibody and C4.

### Increased interhemispheric FC as a compensatory mechanism for reduced brain volume in patients with SLE

Compared with the HC group, the patients with SLE showed significantly lower grey matter volume while increased VMHC of insula and parahippocampal gyrus termed as stronger interhemispheric FC. Due to the atrophy of bilateral insula and parahippocampal gyrus, the interhemispheric communication increase for maintaining normal brain function, reflecting strong reserve ability and plasticity of brain. This compensatory mechanism had also been mentioned in several studies. A previous task-fMRI study of patients with SLE showed greater activation in the insular cortex and dorsolateral prefrontal cortex, while lower activation in the ventromedial prefrontal cortex and cingulate cortex,^19^ revealing a compensatory mechanism was involved in the pathologic process underlying SLE. Studies related to other diseases, such as Alzheimer’s disease (AD) and diabetes mellitus type 2 (T2MD), also found that decrease grey matter volume were accompanied by increased functional activity,^20 21^ which was consistent with our findings. At present, few researchers have evaluated the ability of mirror information transmission between bilateral cerebral hemispheres in patients with SLE. The study by Wang *et al*^22^ found that patients with SLE showed decreased VMHC values in the several gyri when compared with HC group, which confirmed that brain function changes in patients with SLE occurred not only within hemisphere but also between the cerebral hemispheres. Although the VMHC results of this study were different from those of Wang *et al*, we suggested that increased FC might be a compensatory mechanism of brain atrophy.

### Cognition was associated with greater FC and lower brain volume in patients with SLE

The results of this study showed that there were significant correlation between decreased grey matter volume in right insular lobe, increased VMHC in insular lobe and increased TMT duration in patients with SLE. The insula is an essential part of the brain, which grows slowly and is hidden in the lateral sulcus due to its unique growth pattern and different migration trajectories of neurons.^23^ The insula is a crucial component of the salience network, which is involved in complex functions and plays an important role in identifying and integrating high-level cognition, social, emotional processing and sensorimotor processing.^24 25^ Sensory signals are primarily processed in the posterior part of the insula, and then are transmitted to the front part to integrate emotional, cognitive and motivational signals together with the amygdala, anterior cingulate cortex, dorsolateral prefrontal cortex and ventral striatum.^26^ Attention is part of the cognitive process, allowing the brain to strengthen the processing of information. Previous research about the attention capture model of emotional stimulation has revealed that BOLD signal changes of the insula was associated with reaction time,^27^ which revealed that insula was closely associated with attention function. This study showed that the increased VMHC in insula was positively correlated with TMT duration. We still suspected that this was a compensatory mechanism for the impairment of insula to maintain normal cognitive function. A previous study about resting-state brain networks found that patients with SLE exhibited increased FC in the left insular cortex within significant networks and negative correlation with the severity of depression.^28^ Lin *et al* found a positive correlation between cerebellar activity and SLEDAI Score,^29^ interpreted as the mechanism of activation of neural compensation. In our study, the increased VMHC in the insula of patients with SLE was positively correlated with dysfunction of orientation, which was similar to a research reported by Rocca *et al.* They found a significant positive correlation between relative activation in the somatosensory area and the severity of brain damage,^30^ which also suggested the early compensatory effect of neurons. However, the negative correlation between insular grey matter volume and abstract function still needs more studies to explore the underlying mechanisms.

The parahippocampal gyrus is located at the medial side of occipital and temporal lobes, as a main cortical input of hippocampus, which is associated with advanced functions such as learning, emotion and memory.^31^ Our study results revealed that grey matter volume was decreased in the bilateral parahippocampal gyri while the VMHC was increased, which was similar to previous studies. Previously, researchers analysed the dynamic low frequency amplitude (dALFF) of SLE with and without CI, and they found that SLE group with CI exhibited increased dALFF in the right parahippocampal gyrus.^32^ Liu *et al* found that patients with SLE had increased regional homogeneity (ReHo) values in the left parahippocampal gyrus compared with HC.^33^ The opposite functional and structural outcome might suggest compensatory mechanisms in parahippocampal gyrus of patients with SLE. However, whether the dysfunction of parahippocampal gyrus is the neuropathological mechanism of cognitive change in patients with SLE still needs further investigation.

### Relationship between VMHC, grey matter volume changes and serum biochemical markers in the SLE and HC groups

High-affinity anti-dsDNA antibodies are a specific immune indicator for SLE diagnosis. The specificity of anti-dsDNA antibodies for SLE is as high as 90%, which is one of the baseline term in the 2019 SLE classification criteria.^12^ An animal research study on mice found that the insula could store immune information and retrieve specific immune responses under different inflammatory conditions.^34^ Previous studies had shown that blood–brain barrier damage in patients with SLE might occur earlier than obvious neuropsychiatric symptoms,^35^ and then a variety of autoimmune antibodies could penetrate the damaged blood–brain barrier into brain and cause neuronal dysfunction.^36^ Anti-dsDNA antibodies can cross-react with the N-methyl-D-aspartate receptor brain-reactive antibodies, attacking neurons after damaging the blood–brain barrier and is believed to play an important role in the pathogenesis of NPSLE.^37^ Our results showed that in patients with SLE, elevated anti-dsDNA antibody level was associated with decreased VMHC in the insula and parahippocampal gyrus, and higher level of anti-β2GP1 antibody was correlated with lower connectivity in the bilateral insula and more pronounced grey matter atrophy in the left insula. Preziosa *et al*^38^ found that patients with SLE with positive expression of anti-dsDNA antibodies exhibited significant abnormalities in the structural global network properties, as well as prominent reductions in the strength and clustering coefficient of structural hubs. A prospective study tried to predict the neuropsychiatric symptoms in SLE and reveal that anti-dsDNA antibodies could predict multiple neuropathies and psychiatric disorders.^39^ As for our study, we considered that anti-dsDNA antibodies might be an important pathological factor for the brain damage in SLE, and the insula might be a potential target.

Ischaemia is considered as an important pathogenic mechanism of NPSLE. Anti-aPL antibodies can result in endothelial dysfunction, finally leading to thrombosis. Anti-β2GP1 antibody is one marker of the anti-aPL antibodies, and its sustained expression is a characteristic of antiphospholipid syndrome (APS).^40^ Changes in the conformation and post-translational modification of β2GP1 can reduce the protective activity of endothelial nitric oxide synthase and activate the complement, promoting the formation of venous and arterial thrombosis.^41^ A new research has found that targeting domains 1 and 4/5 of β2GP1 was one of the most specific targets for APS, which was helpful for the diagnosis of APS.^42 43^ A retrospective analysis showed that transiently high or low titers of anti-β2GP1 had good predictive value for thrombosis in patients with SLE.^44^ Our study revealed a strong correlation between anti-β2GP1 and grey matter volume in the left insula, as well as insular VMHC, suggesting that anti-β2GP1 might be pathogenic antibody in NPSLE.

In conclusion, our study results further confirmed the abnormal connectivity between brain hemispheres and changes in grey matter volume that were involved in the process of brain damage in SLE. Our findings contributed to revealing the neurobiological mechanisms of NPSLE. The enhanced interhemispheric communication between the insula and parahippocampal gyrus compensated for functional impairments caused by grey matter atrophy. Anti-dsDNA antibodies and anti-β2GP1 might be important serological markers for brain damage in SLE.

### Study limitations

First, this study was a cross-sectional study, so we did not assess the dynamic changes in different brain regions and related antibodies in patients with SLE at different stages. Future longitudinal studies are needed to confirm our findings and further explore their relationship. Second, the lack of serological autoimmune antibodies in HCs is another limitation of our study. In the subsequent study, we will examine serological markers of HCs, such as autoantibodies and other biochemical indicators, in order to improve the quality of our study. Finally, the sample size of this study was small, and we were unable to further subgroup patients with SLE based on the presence of neuropsychiatric symptoms.

## supplementary material

10.1136/lupus-2024-001307 online supplemental file 1

## Data Availability

Data are available on reasonable request.
