# Supplementary material for: Altered structural and functional homotopic connectivity associated with cognitive changes in SLE
Source: Lupus Sci Med. 2024 Nov 24;11(2):e001307. doi: 10.1136/lupus-2024-001307 (PMC11590855; doi:10.1136/lupus-2024-001307)
Supplement: online supplemental file 1 [file lupus-11-2-s001.pdf]

Methods

1.Exploring the imaging specific targets of early brain injury in SLE patients

In this study, SLE patients admitted to the Rheumatology and Immunology Department of the Affiliated Hospital of Inner Mongolia Medical University and healthy controls matched by gender, age and education level were collected. SLE patients were divided into SLE group with neuropsychiatric symptoms (NPSLE) and SLE group without neuropsychiatric symptoms (non-NPSLE). fMRI imaging was performed on all subjects, and analysis of variance was used to compare the functional changes of the three groups, so as to construct a specific imaging model of early brain injury in SLE patients.

2.Searching for risk factors for early brain damage in lupus

Clinical data and blood biochemical immune markers related to SLE patients were collected to further compare serological markers in the NPSLE group and the non-NPSLE group and obtain markers with statistical differences. Multiple regression analysis was constructed between the combined groups to compare the neurocognitive assessment with the different brain regions. To analyze the risk factors that may lead to early brain injury in SLE patients, and provide reference for early diagnosis and treatment of NPSLE.

Inclusion criteria

1.SLE with neuropsychiatric symptoms

(1)Neuropsychiatric symptoms meeting the 1999 ACR definition

(2)Ages 18-55y

(3)Female

(4)Right-handed

(5)Can cooperate with MRI and neuropsychiatric examination

2.SLE without neuropsychiatric symptoms

(1)Patients who meet the 2019 ACR diagnostic criteria for SLE

(2)Patients without neuropsychiatric symptoms

(3)Age 18-55y

(4)Female

(6)Right-handed

(7)Able to cooperate with MRI and neuropsychiatric examination

3.Healthy control group

(1)Age and gender matched the case group

(2)Right-handed

(3)No cerebrovascular, neurological or mental diseases

(4)Can cooperate with MRI and neuropsychiatric examination

Exclusion Criteria:

(1)Patients with serious diseases of other systems

(2)Patients with diseases of central nervous system

(3)Other rheumatic immune system diseases

(4)Neuropsychiatric history

(5)Smokers, drug users or alcoholics

(6)Contraindicated MRI

neuropsychological test

The subjects were evaluated for memory, cognition, mood and sleep

Statistical analysis

SPSS25 and SPM were used for statistical analysis. Analysis of general demographic and neuropsychological tests: one-way ANOVA was used for measurement data conforming to normal distribution, Kruskal-Wallis H test was used for non-normal distribution, and chi-square test was used for counting data to explore differences among the three groups in general data. Based on the MATLAB platform, SPM12 and DPABI were used to analyze the imaging data of the three groups to find the similarity and specificity of brain function changes. Finally, Spearman/Pearson correlation analysis was performed on brain changes, blood biochemical immune markers and cognitive assessment scores in the case group.
